# The importance of taste on swallowing function

**DOI:** 10.3389/fnut.2024.1356165

**Published:** 2024-02-07

**Authors:** Masahiko Okubo, Motoyoshi Morishita, Tomoko Odani, Hideo Sakaguchi, Takeshi Kikutani, Shoichiro Kokabu

**Affiliations:** ^1^Department of Dentistry and Oral Surgery, Ongata Hospital, Hachioji, Tokyo, Japan; ^2^Department of Physical Therapy, Faculty of Rehabilitation, Reiwa Health Sciences University, Fukuoka, Japan; ^3^Department of Dentistry, Kawaguchi Cupola Rehabilitation Hospital, Kawaguchi, Saitama, Japan; ^4^Department of Dentistry, Ryohoku Hospital, Hachioji, Tokyo, Japan; ^5^Division of Clinical Oral Rehabilitation, Nippon Dental University Graduate School of Life Dentistry, Iidabashi, Tokyo, Japan; ^6^Division of Molecular Signaling and Biochemistry, Kyushu Dental University, Kitakyushu, Japan

**Keywords:** aspiration pneumonia, dysphagia, carbonated thickened drink, taste receptors, skeletal muscle, taste, preferences

## Abstract

The world’s population is aging. Pneumonia is the leading cause of death among the older adults, with aspiration pneumonia being particularly common. Aspiration pneumonia is caused by a decline in swallowing function. Causes can include age-related sarcopenia of swallowing muscles, cognitive decline, cerebrovascular and other diseases or even changes in individual taste preference. Currently, the main treatment approach for dysphagia is resistance training of swallowing-related muscles. This approach has not been effective and establishment of novel methods are required. In this review, we introduce and discuss the relationship between taste, taste preference, carbonation and swallowing function. Taste and preference improve swallowing function. Recently, it has been shown that a carbonated beverage that combines the functionality of a thickening agent, the appeal of taste, and the stimulation of carbonation improves swallowing function. This may be very useful in the recovery of swallowing function. It is important to note that deliciousness is based not only on taste and preference, but also on visual information such as food form. Umami taste receptors are expressed not only in taste buds but also in skeletal muscle and small intestine. These receptors may be involved in homeostasis of the amino acid metabolic network, i.e., the process of amino acid ingestion, intestine absorption, and storage in skeletal muscle. Proper stimulation of umami receptors in organs other than taste buds may help maintain nutritional status and muscle mass. Umami receptors are therefore a potential therapeutic target for dysphagia.

## 1 Introduction

Throughout life, the ability to eat well and savor a meal remains one of the most important factors in pursuing happiness. Therefore, developing proper swallowing functions in childhood and maintaining them throughout life is essential.

In 2017, 1.13 million people over the age of 70 (261/100,000) died from secondary community-acquired pneumonia (CAP), a 9% increase in that mortality rate over the past 30 years (1). CAP in the elderly population frequently stems from aspiration pneumonia (AP). Aspiration pneumonia has been proposed as a physiologic phenomenon caused by aspiration due to the age-related decline in swallowing function and cough reflex (2). Furthermore, stroke and prolonged bed rest have been identified as risk factors for aspiration. However, there are still no uniform global diagnostic criteria for aspiration pneumonia. Certain cultures regard and treat aspiration pneumonia in cases of obvious aspiration associated with head and neck cancer or stroke. However, those associated with senility are not considered for active intervention. The proportion of aspiration pneumonia among pneumonia cases that result in hospitalization increases with age, and non-aspiration pneumonia is less common among older adults (3, 4). In other words, aspiration pneumonia poses a significant challenge that must be addressed to ensure healthy longevity in today’s aging society worldwide.

In this review, we would like to discuss the role of taste sensation in swallowing motor functions, their disorders, and the functions of taste receptors expressed outside the oral mucosa. We can examine strategies for overcoming swallowing disorders, paving the way for a healthy and fulfilling life throughout our entire lifespan.

## 2 Population aging worldwide and dysphagia/aspiration pneumonia

Swallowing disorders are characterized and classified by the following: [1] Inability to form a bolus of food in the oral cavity and to transport the bolus to the pharynx. [2] Misdirection of food and fluid in the pharynx, causing it to enter the trachea instead of the esophagus. [3] Inability to get rid of food or liquid accidentally entering the trachea by coughing (5).

In today’s aging society, aspiration pneumonia due to dysphagia in older adults is becoming an increasing problem (6). Older adults over 65 years old account for two-thirds of those affected by dysphagia (7). In the United States, aspiration pneumonia was reported as the underlying cause of death in an average of 17,616 cases per year, representing 30.1% of all aspiration pneumonia-related deaths. Individuals aged 75 and over accounted for 76.0% of deaths from aspiration pneumonia, with an age-adjusted rate ratio of 161.0 (CI 160.5–161.5) (8). Maintaining oral hygiene and swallowing function is important in preventing aspiration pneumonia (9).

## 3 Swallowing and skeletal muscle

Various muscles are involved in swallowing movements. They include the masticatory muscles, the tongue, and the perifacial muscles involved in mastication movements. They include the supralaryngeal muscles involved in elevating the larynx. The sublingual muscles support the hyoid bone, and the pharyngeal contractile muscles increase swallowing pressure. However, this is not the only cause of dysphagia, as poor posture with aging, such as hunchback and neck anteflexion, can lead to compensatory use of muscles needed for swallowing, resulting in secondary dysphagia (10).

Although sarcopenia is a disease characterized by generalized muscle loss, muscle weakness, and loss of physical function, the muscles associated with swallowing do not seem to lose muscle mass and strength like other muscles. These muscles, except the geniohyoid muscle, are actively engaged in respiration, even at rest, under the control of the respiratory center. Therefore, it has been thought that disuse muscle atrophy is unlikely to occur in the muscles associated with swallowing (11). In contrast, there are reports of accelerated muscle atrophy of the tongue and diaphragm in patients with aspiration pneumonia (12), and studies show that muscle atrophy occurs in the respiratory muscles and muscles associated with swallowing (13).

Various types of training are available to improve swallowing function, including resistance training of swallowing-related muscles (14–16) and neuroelectrical stimulation therapy (17).

## 4 Taste receptors and skeletal muscle

The Tas1R family (Tas1r1, Tas1r2, and Tas1r3) are G protein-coupled receptors that sense sweet taste in the Tas1r2/Tas1r3 complex and umami taste in the Tas1r1/Tas1r3 complex as taste receptors. Recent studies have revealed that the Tas1R family members function as nutrient sensors in tissues other than the oral mucosa (18).

Skeletal muscle is one of the largest organs in the human body, accounting for approximately 40% of body mass. With age, skeletal muscle mass tends to decrease, leading to a condition known as sarcopenia. Sarcopenia is characterized by muscle weakness and decreased physical activity capacity, which can significantly impact the quality of life for older adults. Since muscle is a central organ that takes up and consumes sugar and fatty acids from the blood, its maintenance is considered essential for preventing metabolic-related diseases such as obesity and diabetes (19).

Satellite cells are muscle stem cells that provide a regenerative capacity to skeletal muscle. Aged skeletal muscles have an impaired regenerative capacity which can contribute to physical incapacitation. Aged skeletal muscles fail to retain stem cell quiescence (20–22). Muscle stem cell number and the functionality decline with aging (20–25). The process of autophagy, which involves the degradation of long-lived proteins and damaged organelles in lysosomes, has been implicated in the aging of different model organisms (22, 26–29). Maintenance of skeletal muscle mass also relies on the balance between anabolic and catabolic processes. Protein degradation in skeletal muscle cells is essentially mediated by the activity of two conserved pathways: the ubiquitin proteasomal pathway and the autophagic/lysosomal pathway (30). The ubiquitin-proteasomal pathway is responsible for the turnover of the majority of soluble and myofibrillar muscle proteins (31, 32). Autophagy also plays an important role in the degradation of skeletal muscle (33).

Tas1r1 and Tas1r3, are highly expressed in muscle relative to other tissues (34). We reported that myogenic regulatory factor (MRF)s regulate the expression of Tas1r3 (35). Overexpression of MyoD and Myogenin induces murine Tas1r3 promoter activity. ChIP analysis demonstrated that MyoD and Myogenin bind to the endogenous murine Tas1r3 promoter and increase mRNA levels of endogenous Tas1r3 in murine myoblasts. We demonstrated that the expression of Tas1r1 also increased during myogenesis in a cell culture model (36). These findings are further supported by the observation that Tas1r1 and Tas1r3 are endogenously expressed in skeletal muscle tissue. The skeletal muscle Tas1r3 knockout mice exhibit decreased activity of the mammalian target of rapamycin complex 1 (mTORC1) and a higher frequency of autophagy (34), suggesting that umami receptor function is critical to detecting nutrient status since skeletal muscle is the main source of stored amino acid during times of amino acid deprivation (37). For these reason, we hypothesized that disorder of signaling through Umami receptor is involved in pathogenesis of skeletal muscle related diseases including sarcopenia and swallowing disorders.

## 5 Rehabilitation, nutrition, and taste

Adequate nutrition is essential for enhancing the effectiveness of rehabilitation through strength training, and the concept of rehabilitation nutrition is gaining increasing recognition (38). The importance of the trinity of rehabilitation, nutrition, and oral management for sarcopenic dysphagia in older adults is emphasized (39). Taste is essential to sustain life, and its primary function is to facilitate the intake of essential nutrients and the rejection of harmful substances. Umami receptors are expressed on epithelial cells in the small intestine. Glutamate stimulation via umami receptors in the small intestine is essential for maintaining the normal turnover of small intestinal epithelial cells (40). Insufficient umami stimulation of umami receptors in the small intestine can lead to impaired nutrient absorption from the small intestine. To ensure optimal nutrient intake, it is necessary to study the sense of taste and the regulatory mechanism of nutrient absorption via taste receptors in the intestinal tract.

## 6 Swallowing function and sense of taste and preferences

Swallowing function is known to be influenced by individual preferences. High temporal resolution fMRI studies show that taste, smell, and visual sensations stimulated by highly palatable foods, such as popcorn and chocolate, activate areas of the cerebral cortex associated with swallowing (sensorimotor area, insular cortex, cingulate gyrus, prefrontal cortex) (41). Taste stimuli alter the swallowing reflex. Taste, particularly acidity, increases swallowing pressure and suprahyoid muscle activity (42–44). Other reports indicate that salty and sweet stimuli increase swallowing pressure and suprahyoid muscle activity (43–45). However, foods with intense sour or salty flavors that directly trigger swallowing are considered problematic regarding taste and palatability (43). Conversely, there is a positive correlation between taste and swallowing function. Salty and sweet stimuli are more effective than no-taste stimuli in increasing swallowing pressure and supratrochlear muscle activity (42, 44, 45). A study involving young, healthy subjects compared the ease of swallowing foods with the five basic tastes (sweet, salty, sour, bitter, and umami) against tasteless foods. Sweet and tasteless foods exhibited slightly better swallowing acceptability than by sour and bitter foods (46). Investigations involving healthy adult male volunteers have shown that cortical swallowing pathways are similarly modulated by both sweet and bitter stimuli (46). This study implies the existence of a close interaction between taste perception and swallowing activity via the central nervous system.

## 7 Swallowing function-carbonation and taste

Swallowing movements are not merely simple muscle contractions; rather, they are controlled by various nerves. Stimulating these sensory nerves is important for maintaining their functionality and overcoming swallowing dysfunction. Using carbonated beverages as a stimulatory modality has recently garnered attention for its efficacy in enhancing swallowing movements. Carbonated water increases muscle activity during swallowing more than plain water (47), and the higher gas volume of carbonated water increases tongue pressure (48). In addition to its effect on muscle activity, carbonated water improves pharyngeal clearance with significantly faster pharyngeal transit time compared to normal liquids (49). Furthermore, carbonated water supports the shortening of tablet transit time during tablet swallowing (50). Furthermore, carbonated water is less likely to be aspirated or left in the pharynx than non-carbonated liquids in patients with central nervous system disease (51, 52). We and other laboratories have reported that thickened carbonic acid further improves swallowing function (53, 54).

As one of the reasons why carbonated beverages improve swallowing, H_2_CO_3_ produced by carbonic anhydrase IV in saliva from CO_2_ dissolved in carbonated beverages stimulates nociceptors in the oral mucosa and activates the trigeminal nerve (55). In addition, the carbonic acid in carbonated water splits into bicarbonate and hydrogen ions, and the hydrogen ions stimulate the facial nerve via acid-sensitive taste receptors (56). Stimulation with capsaicin improves swallowing function (57).

Capsaicin-induced improvement of swallowing involves TRPV1 at the nerve terminal. ASIC3 is a receptor expressed at nerve endings similar to TRPV1. The ASIC3 receptor has been implicated in the effects of carbonic acid stimulation on swallowing (58).

In contrast, there are reports that swallowing only carbonated water can worsen the penetration-aspiration scale (PAS) and videofluoroscopic dysphagia scale (VDS) (59, 60). This is thought to be influenced by preference and taste (Please see preceding paragraph).

## 8 Swallowing and food forms

The available forms of food intake are frequently restricted due to poor swallowing functions. However, even if dysphagia-adjusted diets, such as kizami or paste diets, are suitable for the remaining swallowing functions, these dysphagia-adjusted diets do not maintain the original food form and may decrease the patient’s desire to eat (61). Sight and smell control appetite (62). Other laboratories reported that the arrangement of coloring, smell, and flavor could increase food intake and improve nutritional status, while dysphagia-adjusted diets serve as food form (63–65). Eating well brings a sense of spiritual richness and satisfaction, and it helps establish and maintain social relationships and communication. Because social activities are often limited for older adults, meals are a major recreation component.

Yoshihara et al. (66) reported a positive correlation between appetite and quality of life in a study of an elderly community in Japan. Eating food that is not tasty solely for sustenance can be a disagreeable experience. Meals should be enjoyable to consume in terms of flavor and presentation. Older individuals exhibit different eating patterns compared to young children just starting to learn regarding food. Older individuals have had the pleasure of enjoying many delicious meals throughout their lives. The purpose of a meal extends beyond simply fulfilling nutritional and energy needs. The European Pressure Ulcer Advisory Panel’s guidelines for pressure ulcer prevention and treatment include considerations for appetizing food with attractive presentations (67).

## 9 Discussion

**Figure 1:**
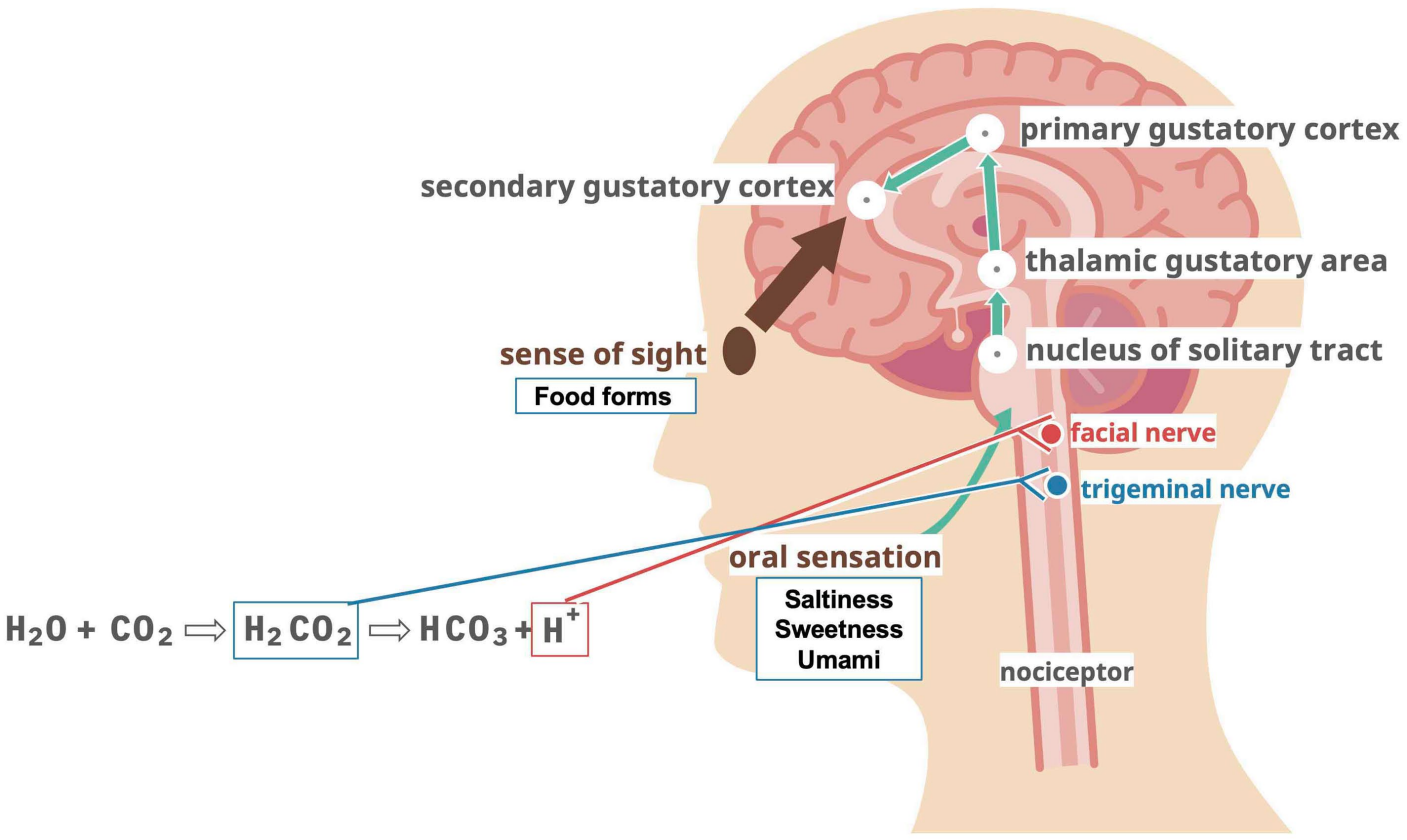
Hypothesized mechanisms of action for taste, vision, and carbonic acid stimulation.

Thus, swallowing function is closely related not only to mastication and pharyngeal muscle contraction but also to various stimulus inputs (Figure 1). Therefore, simple muscle training may not be sufficient to treat dysphagia. This review focused on swallowing functions regarding taste and taste receptors. Maintaining swallowing functions and taking adequate nutrients are necessary to maintain swallowing functions. On the contrary, poor nutritional status naturally leads to a decline in swallowing function. This is indeed a chicken and egg situation between “swallowing” and “nutrition.”

**Figure 2:**
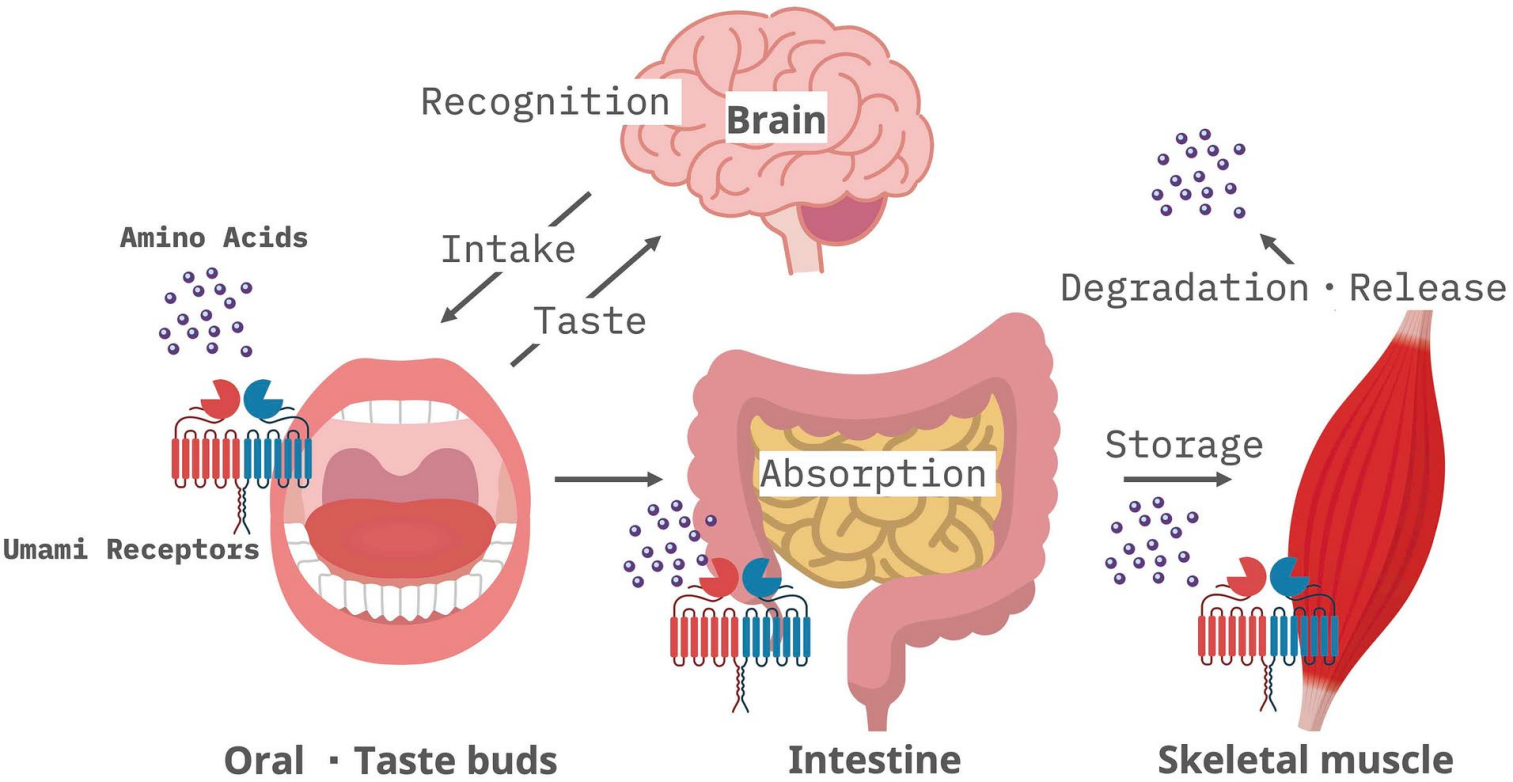
Amino acid network via umami receptors expressed by oral mucosa, intestine, and skeletal muscle.

Amino acids (proteins) in food are received by umami receptors (Tas1r1/Tas1r3) in the taste buds of the oral mucosa. These amino acids (proteins) are recognized as “delicious” taste sensations and are actively absorbed after digestion and stored in skeletal muscles as myofiber proteins. Muscle fibers are depleted during starvation, and amino acids are released into the bloodstream (37). In sarcopenia, this amino acid network (Figure 2) may be disrupted, leading to the breakdown of skeletal muscle proteins. This releases more amino acids that are taken up, causing a decrease in skeletal muscle mass and function. On the other hand, Tas1r3 knockout does not alter amino acid concentrations due to compensatory increases of other amino acid transporters (34). In addition to this, there are several issues that need to be resolved before deciding the function of taste receptor in skeletal muscle. For example, do Tas1r1 and Tas1r3 form a heteromeric complex in muscle cells similar to in taste bud cells? What is the ligand for taste receptors when they function as amino acid sensors in skeletal muscle? Careful *in vivo* experimental analysis focusing on skeletal muscle metabolism will be required to address these issues.

Humans with decreased sensitivity to umami taste were reported to show significant weight loss and deterioration in general health compared to those with other taste disorders (sweet, salty, sour, and bitter) (68). In this report, it was interpreted that the patients had difficulty perceiving umami, which may have made the food less palatable and reduced their food intake. Moreover, a reduction in the expression level and sensitivity of umami receptors at the genomic level diminishes taste perception and impairs the function of umami receptors in the small intestinal mucosa, skeletal muscle, and other organs that comprise the amino acid sensing network. In other words, the observed weight loss and decline in overall health may be manifestations of underlying nutritional deficiencies caused by impaired digestion and absorption and reduced skeletal muscle mass resulting from abnormal skeletal muscle metabolism. Multiple single nucleotide polymorphisms are observed in the gene encoding the umami receptor, with each mutation having a different sensitivity to the umami receptor *in vitro* (69). Therefore, based on their genetic background, humans have reduced sensitivity to umami receptors in the body.

Medical advancements have significantly extended our lifespans, allowing us to delay death’s inevitable arrival. However, this progress has created a disparity between healthy life expectancy, the number of years an individual can expect to live independently in society, and overall life expectancy, which simply marks the end of life. In addition, the collapse of current healthcare systems and the economic disparity in the selection of life are becoming realities due to the extremely expensive drugs created by the latest life science and technology. If we can advocate an inexpensive extension of healthy life expectancy based on food, we can solve these problems and make a major shift toward a sustainable super-aging society.

This review article highlights the association between swallowing disorders and sarcopenia of the associated muscles. Diseases that cause skeletal muscle atrophy, such as sarcopenia, significantly reduce healthy life expectancy. Currently, no commercialized interventions, except for exercise, exist to maintain or restore skeletal muscle mass and function. Considering the high prevalence and pathogenesis of sarcopenia, the prevention and treatment of sarcopenia should be based on a healthy diet combined with adequate exercise rather than expensive medicine such as molecularly targeted drugs. Sufficient amino acid intake, a crucial component of skeletal muscle, is essential for preventing sarcopenia. Future research endeavors to identify amino acid combinations that efficiently activate the umami receptors of each cell in the amino acid network may contribute to overcoming sarcopenia and thereby extending healthy life expectancy in this super-aging society.

## Author contributions

MO: Writing – original draft, Writing – review & editing. MM: Writing – review & editing. TO: Writing – review & editing. HS: Writing – review & editing. TK: Writing – review & editing. SK: Conceptualization, Writing – original draft, Writing – review & editing.
